# Vitamin D Levels and Lipid Response to Atorvastatin

**DOI:** 10.1155/2010/320721

**Published:** 2009-08-19

**Authors:** José Luis Pérez-Castrillón, Laura Abad Manteca, Gemma Vega, Javier del Pino Montes, Daniel de Luis, Antonio Dueňas Laita

**Affiliations:** ^1^Department Internal Medicine, Rio Hortega Universitary Hospital, C/ Dulzaina 2, University of Valladolid, 47012 Valladolid, Spain; ^2^RETICEF, Spain; ^3^Department of Medicine, Clinic Hospital, University of Salamanca, Spain

## Abstract

Adequate vitamin D levels are necessary for good vascular health. 1,25-dihydroxycholecalciferol activates CYP3A4, an enzyme of the cytochrome P450 system, which metabolizes atorvastatin to its main metabolites. The objective of this study was to evaluate the response of cholesterol and triglycerides to atorvastatin according to vitamin D levels. Sixty-three patients with acute myocardial infarction treated with low and high doses of atorvastatin were included. Levels of total cholesterol, triglycerides, HDL cholesterol, and LDL cholesterol were measured at baseline and at 12 months of follow-up. Baseline levels of 25-hydroxyvitamin D (25-OHD) were classified as deficient (<30 nmol/L), insufficient (30–50 nmol/L), and normal (>50 nmol/L). In patients with 25-OHD <30 nmol/L, there were no significant changes in levels of total cholesterol (173 ± 47 mg/dL versus 164 ± 51 mg/dL), triglycerides (151 ± 49 mg/dL versus 177 ± 94 mg/dL), and LDL cholesterol (111 ± 48 mg/dL versus 92 45 ± mg/dL); whereas patients with insufficient (30–50 nmol/L) and normal vitamin D (>50 nmol/L) had a good response to atorvastatin. We suggest that vitamin D concentrations >30 nmol/L may be required for atorvastatin to reduce lipid levels in patients with acute myocardial infarction.

## 1. Introduction

Statins, HMG-CoA reductase inhibiters, are effective in the primary and secondary prevention of cardiovascular disease and act principally by reducing cholesterol and triglyceride levels. The relationship between high levels of plasma cholesterol and atherosclerotic vascular disease is clearly established, with a reduction in total cholesterol and LDL cholesterol to below critical levels significantly reducing the risk [1, 2]. The limits for HDL cholesterol and triglycerides are not so clearly established. Statins are first-line agents for the prevention of vascular risk, with new drugs being added when the objectives established by guidelines are not reached.

Vitamin D, in addition to increasing intestinal calcium absorption, reducing parathyroid hormone levels and improving the amount and quality of bone, has a beneficial vascular effect. Vitamin D deficiency has been associated with peripheral arterial disease and myocardial infarction [3, 4]. Vitamin D deficiency may increase the risk of cardiovascular disease through three possible mechanisms. Firstly, in vitro studies have shown that vitamin D may cause an imbalance between anti-inflammatory and proinflammatory cytokines, reducing NF-*κ*B activity, increasing IL-10 production, and reducing levels of IL-6, IL-1, interferon *γ*, and TNF*α* [5]. Secondly, endothelial cells have receptors for vitamin D whose stimulation inhibits cell proliferation [6]. Thirdly, the association between hypertension and vitamin D deficit is mediated by activation of the renin-angiotensin-aldosterone system. High calcitriol levels reduce plasma renin activity, leading to reduced plasma angiotensin II concentrations. This modulation of the renin-angiotensin-aldosterone system, in addition to reducing blood pressure, reduces inflammation of the vascular endothelium, thus limiting atherosclerosis progression [7]. Therefore, adequate vitamin D levels are necessary for good vascular health.

In addition, 1,25-dihydroxycholecalciferol, the active metabolite of vitamin D, bonds with the vitamin D receptor, activating CYP3A4, an enzyme of the cytochrome P450 system, which metabolizes atorvastatin to its main metabolites [8]. Therefore, low vitamin D levels might reduce CYP3A4 activity, increasing atorvastatin levels and making it more effective in reducing cholesterol and triglyceride levels.

The objective of this study was to evaluate the response of cholesterol and triglycerides to atorvastatin according to vitamin D levels.

## 2. Methods

Consecutive patients hospitalized for acute *myocardial infarction* were eligible for inclusion. Patients were recruited at hospital admission. Exclusion criteria were alcoholism, neoplasia, hyper- or hypocalcemia, and treatment with vitamin D, *alendronate*, *risedronate*, *zoledronate*, *raloxifene*, *PTH* *1-34*, *strontium* *ranelate*, or *estrogens*. After diagnosis, patients received low (10–20 mg) or high doses (40–80 mg) of atorvastatin as secondary prevention according to baseline levels of cholesterol and triglycerides and the index of vascular risk in accordance with the Score chart calibrated for the Spanish population [9]. Only patients completing follow-up were evaluated. Patients were classified according to levels of 25-hydoxyvitamin D (25-OHD) as deficient (25-OHD <30 nmol/L), insufficient (30 nmol/L–50 nmol/L), and normal (>50 nmol/L) [10].

Blood samples were obtained after 8 hours of fasting in the first three days after admission. Cholesterol, triglyceride, HDL cholesterol, and phosphatase levels were measured using a Hitachi 917 autoanalyser (Tokyo, Japan). LDL cholesterol was calculated by an indirect method. 25-OHD levels were determined by high-performance liquid chromatography with a 12% interassay variation coefficient.

A descriptive statistical analysis was made, including measures of central tendency and scattering for quantitative variables. The Student's *t*-test was used to compare variables and the Anova test for multiple comparisons. Results are expressed as mean ± standard deviation; the level of statistical significance was established as *P* = .05. The study was approved by the clinical research committee of the hospital and all patients gave written informed consent to participate in the study.

## 3. Results and Discussion

Sixty-three patients with acute myocardial infarction (40 males and 23 females) with ages ranging between 37 and 79 years were included. All females were postmenopausal. 25-OHD levels at baseline were <30 nmol/L in 13 patients, 30–50 nmol/L in 34, and >50 nmol/L in 16 patients. Baseline patient characteristics showed no significant differences between groups, although 76% of patients with 25-OHD <30 nmol/L have received high doses of atorvastatin. There were no significant differences between the three groups with respect to the distribution of the doses (Table 1).

Table 2 shows the response to atorvastatin at 12 months according to vitamin D levels. In patients with 25-OHD <30 nmol/L, there was no reduction in levels of total cholesterol, triglycerides, and LDL cholesterol but there was a significant rise in levels of HDL cholesterol. Patients with insufficient and normal vitamin D levels had the expected response, with a reduction in levels of total cholesterol, triglycerides, LDL cholesterol, and elevation of HDL. There were no differences in the response according to gender or the dose of atorvastatin used, with total cholesterol levels falling in patients receiving high doses (181 ± 49 versus 157 ± 39, *P* = .0008) and low doses (188 ± 45 versus 169 ± 41, *P* = .02).

Our results show that the reduction in levels of cholesterol and triglycerides was significantly greater in patients with insufficient and normal vitamin D levels compared to those with deficient levels. Other authors have shown an added effect of vitamin D, not only on levels of cholesterol and triglycerides but also on the prevalence of cardiovascular disease [11].

The efficacy of atorvastatin depends on its plasma concentrations and those of its hydroxylated metabolites. Atorvastatin has low oral bioavailability due to rapid hepatic and renal metabolization by enzymes of the P450 system, mainly CYP3A4-1 [12]. This system is a nexus of union between statins and vitamin D. Bonding of the active metabolite of vitamin D brings about a change in the conformation of the receptor, which is closely linked to the X receptor of retinoic acid, forming a heterodimer [13]. This activates gene transcription, facilitating the synthesis of specific proteins, including the enzymes of the cytochrome P450 system (CYP3A11, CYP3A1, and CYP3A4) [14]. These enzymes participate in the oxidation of atorvastatin, giving rise to the synthesis of two active metabolites, 2-hydroxyatorvastatin and 4-hydroxyatorvastatin [15]. Vitamin D induces P450, reducing concentrations of atorvastatin and its active metabolites, as shown by Schwartz [16], who studied the effects of vitamin D supplements on atorvastatin concentrations and cholesterol in 16 healthy volunteers. Vitamin D induced CYP3A4, reducing active concentrations of atorvastatin and its metabolites. This reduction should produce an increase in cholesterol and LDL cholesterol levels. However, the inverse was observed, with a fall in total cholesterol and LDL cholesterol levels. These data reflect our findings.

The enzyme, 3-hydro-3-methylglutaryl coenzyme A (HMG-CoA) reductase, plays a key role regulating the synthesis of cholesterol. Hydroxylation of sterols generates a powerful inhibition of reductase activity. Defay et al. [17] found that hydroxylated vitamin D derivatives inhibited HMG-CoA reductase activity in lymphocytes stimulated with phytohemaglutinin. Experimental studies in various cell lines have shown that treatment with cholecalciferol (vitamin D3) and its metabolites (25-hydroxycholecalciferol, 1,25-dihydroxycholecalciferol, and24, 25- dihydroxycholecalciferol) inhibit cholesterol synthesis due to inhibition of the activity of HMG-CoA reductase, a key enzyme in cholesterol synthesis. This effect is not produced by the active metabolite of vitamin D, 1,25-dihydoxyvitamin D [18]. The inhibition of enzyme activity was concentration dependent. In addition, 25-hydroxyvitamin D3 can inhibits lanosterol 14*α*- demethylase (CYP51A1), an enzyme which also intervenes in the synthesis of cholesterol. A deficit of 25-hydroxycholecalciferol increases both reductase and CYP51A1 activity, raising cholesterol levels. Atorvastatin acts on this enzyme, and therefore it may be hypothesised that a specific concentration of vitamin D is required for it to synergize with the statin and exert its effect on lipid metabolism.

In a previous study, we found an increase in 25-hydroxyvitamin D levels after a year of treatment with atorvastatin, with a reduction in the number of patients deficient in this hormone [4]. Similar findings have been observed in two small studies including 18 patients with familiar hypercholesterolemia, where the administration of statins (lovastatin, simvastatin) resulted in rises in serum concentrations of vitamin D [19, 20]. The mechanism responsible was related to the inhibition of 3-hydroxy-3 methylglutaryl coenzyme A (HMG-CoA) reductase which catalyzes 7-dehydrocholesterol to cholesterol. 7-dehidrocholesterol is a precursor not only of cholesterol but also of vitamin D [21]. Enzyme blockade may divert 7-dehidrocolesterol to the synthesis of cholecalciferol, raising its concentration. This increase might explain part of the beneficial effect of statins on bone metabolism, the increase in bone mineral density, and the reduction in the number of fractures.

These data contribute new insight on the extraskeletal action of vitamin D. Pilz et al. [22] recently showed that vitamin D deficiency was associated with an increase in cardiovascular mortality due to heart failure or sudden death in 3229 patients referred for coronary angiography. After adjusting for vascular risk factors, patients with vitamin D <25 nmol/L had a threefold greater risk of death due to heart failure and a fivefold greater risk of sudden death during the seven-year follow-up. Other reports show similar results [23].

## 4. Conclusion

Although the study sample is small, our results suggest that plasma 25-OHD concentrations above 30 nmol/L are required for atorvastatin to reduce levels of total cholesterol, triglycerides, and LDL cholesterol in patients with acute myocardial infarction.

## Figures and Tables

**Table 1:** Baseline patient characteristics according to vitamin D levels. High doses: atorvastatin (40–80 mg). Low doses: atorvastatin (10–20 mg).

	Vitamin D <30,	Vitamin D 30–50,	Vitamin D >50,	*P*
	nmol/L (*n* = 13)	nmol/L (*n* = 34)	nmol/L (*n* = 16)	
Age (years)	61 ± 11	61 ± 10	62 ± 10	0.981
Weight (kg)	75 ± 10	75 ± 12	72 ± 10	0.552
Height (cm)	167 ± 8	163 ± 9	164 ± 9	0.482
High doses	10	14	10	0.072
Low doses	3	20	6	0.060
Male/female	9/4	20/14	11/5	0. 706

**Table 2:** Vitamin D levels and response to atorvastatin.

	Vitamin D <30,		Vitamin D 30–50,		Vitamin D >50,	
	nmol/L (*n* = 13)		nmol/L (*n* = 34)		nmol/L (*n* = 16)	
	Baseline	12 m	Baseline	12 m	Baseline	12 m
Cholesterol mg/dL	173 ± 47	164 ± 51	177 ± 49	157 ± 31^b^	202 ± 43	163 ± 25^e^
Triglycerides mg/dL	151 ± 49	177 ± 94	154 ± 121	114 ± 55	157 ± 61	110 ± 37^f^
HDL cholesterol mg/dL	34 ± 6	45 ± 9^a^	36 ± 11	49 ± 13^c^	41 ± 8	50 ± 10^g^
LDL cholesterol mg/dL	111 ± 48	92 ± 45	112 ± 42	86 ± 31^d^	114 ± 29	89 ± 26^h^

(a) *P* = .001, (b) *P* = .021, (c) *P* = .0001, (d) *P* = .01, (e) *P* = .003, (f) *P* = .0001, (g) *P* = .008, (h) *P* = .022.
